# A common variant in the hepatobiliary phospholipid transporter *ABCB4* modulates liver injury in PBC but not in PSC: prospective analysis in 867 patients

**DOI:** 10.1186/s13023-022-02565-6

**Published:** 2022-11-17

**Authors:** Beata Kruk, Malgorzata Milkiewicz, Joanna Raszeja-Wyszomirska, Piotr Milkiewicz, Marcin Krawczyk

**Affiliations:** 1 Laboratory of Metabolic Liver Diseases, Department of General, Transplant and Liver Surgery, Medical University of Warsaw, Warsaw, Poland; 2 Department of Medical Biology, Pomeranian Medical University in Szczecin, Szczecin, Poland; 3 Liver and Internal Medicine Unit, Medical University of Warsaw, Warsaw, Poland; 4 Translational Medicine Group, Pomeranian Medical University in Szczecin, Szczecin, Poland; 5 Department of Medicine II, Saarland University Medical Center, Saarland University, Homburg, Germany

**Keywords:** ATP-binding cassette transporter, Cirrhosis, Liver fibrosis, Primary biliary cholangitis, Primary sclerosing cholangitis, Single nucleotide polymorphism, Phospholipid transporter variant

## Abstract

**Background:**

The ATP-binding cassette subfamily B member 4 (*ABCB4*) gene encodes the hepatic phospholipid transporter. Variants in the *ABCB4* gene are associated with various cholestatic phenotypes, some of which progress to liver fibrosis and cirrhosis. The aim of our study was to investigate the role of the cholestasis-associated variant *ABCB4* c.711A > T (p.I237I, rs2109505) in patients with primary biliary cholangitis (PBC) and primary sclerosing cholangitis (PSC).

**Results:**

Two cohorts of Polish patients took part in this study. The Szczecin cohort comprised 196 patients with PBC (174 females, 38% with cirrhosis) and 135 patients with PSC (39 females, 39% with cirrhosis). The Warsaw cohort consisted of 260 patients with PBC (241 females, 44% with cirrhosis) and 276 patients with PSC (97 females, 33% with cirrhosis). Two control cohorts—150 healthy blood donors and 318 patients without liver disease, were recruited in Szczecin and in Warsaw, respectively. The *ABCB4* c.711A > T polymorphism was genotyped using TaqMan assay. In both PBC cohorts, carriers of the risk variant presented more frequently with cirrhosis (Szczecin: OR = 1.841, *P* = 0.025; Warsaw: OR = 1.528, *P* = 0.039). The risk allele was associated with increased serum AST, GGT and ALP (all *P* < 0.05) at inclusion. During the follow-up, patients in both cohorts significantly improved their laboratory results, independently of their *ABCB4* c.711A > T genotype (*P* > 0.05). During 8 ± 4 years follow-up, a total of 22 patients in the Szczecin PBC group developed cirrhosis, and this risk was higher among carriers of the risk variant (OR = 5.65, *P* = 0.04). In contrast to PBC, we did not detect any association of *ABCB4* c.711A > T with a liver phenotype in PSC cohorts.

**Conclusions:**

The frequent pro-cholestatic variant *ABCB4* c.711A > T modulates liver injury in PBC, but not in PSC. In particular, carriers of the major allele are at increased risk of progressive liver scarring.

**Supplementary Information:**

The online version contains supplementary material available at 10.1186/s13023-022-02565-6.

## Background

Primary biliary cholangitis (PBC) and primary sclerosing cholangitis (PSC) represent two rare chronic cholestatic liver diseases [1]. They can progress to liver fibrosis and cirrhosis and the pathogenesis of both is unclear [2–5]. PBC affects interlobular bile ducts, causing ductopenia and progressive cholestasis [5]. It is characterized by immune-mediated destruction of intrahepatic small bile ducts and lymphocyte—predominant portal inflammation [3, 4]. PSC is characterized by progressive ongoing chronic inflammation and destruction of intrahepatic and extrahepatic bile ducts, hepatocellular damage and fibrosis [2]. The role of genetic predisposition in PBC and PSC has been suspected, but so far genetic studies have yielded inconclusive results [6]. Liver scarring and its consequence—impaired liver function and portal hypertension—affect the health span of patients with chronic liver disease [7]. Since the progression of chronic cholestasis varies between patients, genetic modifiers of this process have also been investigated but to date no major genetic modifiers of PBC and PSC have been identified.

The *ABCB4* (ATP-binding cassette subfamily B member 4) gene, located on chromosome 7q21.1, encodes the hepatobiliary phospholipid transporter [8]. ABCB4, also known as multidrug resistance protein 3 (MDR3 in humans, Mdr2 in mice), is a membrane transport protein, which is expressed almost exclusively in the liver [8]. MDR3 acts as an energy-dependent transporter, moving phospholipids from the inner to the outer leaflet of the canalicular membrane of the hepatocyte [9]. Phospholipids inside bile canaliculi combine with cholesterol and bile salts to create mixed micelles which attenuate the toxic effects of bile on the epithelial cells of bile ducts [10]. Dysfunction of ABCB4 can lead to a range of clinical phenotypes ranging from slightly increased liver function tests, to gallstone disease, intrahepatic cholestasis of pregnancy (ICP), progressive familial intrahepatic cholestasis type 3 (PFIC-3), and biliary cirrhosis [11–13]. Among common *ABCB4* variants, c.711A > T (p.I237I, rs2109505) has previously been associated with the risk of developing ICP [14, 15]. Gudbjartsson et al. analysed sequencing results of 2636 Icelanders and suggested that this variant might represent a general risk factor for liver disease (gallstone disease, gallbladder and bile duct carcinoma, liver cirrhosis, higher serum levels of AST, ALT and GGT) [16]. A recent large-scale whole-genome sequencing study in India revealed a significant association between this variant and the risk of gallbladder cancer [17]. Recently, it was also shown that ABCB4 deficiency might be associated with an impaired quality of life [18].

Here we investigate if the *ABCB4* c.711A > T risk allele enhances liver injury in patients with PBC and PSC. To this end we analysed the effects of this variant on: (1) disease progression (liver cirrhosis, liver transplantation), (2) circulating levels of markers of liver injury, (3) disease progression during follow-up, (4) results of quality of life questionnaires.

## Results

### Clinical characteristics of the study cohorts

Table 1A, B summarize the clinical characteristics of study cohorts. In total 867 Polish patients (456 with PBC and 411 with PSC, 551 women, age range 17–87 years) were included in the study. Among them, 331 were recruited at the Pomeranian Medical University in Szczecin (Table 1A) and 536 at the Medical University in Warsaw (Table 1B). In the Szczecin cohort, 196 patients had PBC (174 women, age range 23–87 years) and 135 had PSC (39 women, age range 17–69 years). The PBC group was characterized by a higher number of cirrhotic patients compared to PSC patients (41%/35%, respectively). As demonstrated in Table 1A, in the cohort from Szczecin, patients with PBC were older, had significantly higher BMI but lower serum hemoglobin, platelet count and alanine aminotransferase (ALT) as compared to patients with PSC. In the Warsaw cohort, 260 patients had PBC (241 women, age range 29–86 years) and 276 patients had PSC (97 women, age range 22–81 years). Compared to patients with PSC, PBC patients were older, of higher BMI and had significantly lower serum alanine aminotransferase (ALT), aspartate aminotransferase (AST), alkaline phosphatase (ALP), gamma-glutamyl transferase (GGT), hemoglobin and platelet counts (Table 1B). The control cohort from Szczecin consisted of 150 healthy blood donors (127 women), the control cohort from Warsaw comprised 318 patients without acute or chronic liver disease (290 women).

**Table 1:** Clinical and laboratory characteristic of PBC and PSC cohorts recruited in (A) Szczecin, (B) Warsaw

Variables	Subject characteristics			*P*
	PBC		PSC	
*(A)*				
N	196		135	
Gender, female/male (%)	174 (89%)/22 (11%)		39 (29%)/96 (71%)	< 0.001
Age (years)	60 (23–87)		35 (17–69)	< 0.001
Age at diagnosis (years)	55 (22–83)		32 (4–66)	
IBD, none/UC/Crohn's disease/undifferentiated colitis	N.a		73/44/4/14	
Cirrhosis, (%)	74 (38%)		53 (39%)	0.217
Hemoglobin (mg/dl)	12.5 (9.0–15.4)		13.1 (7.8–16.7)	0.034
PLT (10^3^/µl)	197 (32–728)		239 (42–721)	0.018
Creatinine (mg/dl)	1.3 (0.4–1.5)		0.7 (0.2–1.5)	0.580
Bilirubin (mg/dl)	2.4 (0.2–29.3)		2.2 (0.2–19.1)	0.493
ALT (U/L)	63 (10–539)		97 (8–678)	0.001
AST (U/L)	67 (13–534)		75 (14–438)	0.290
ALP (U/L)	238 (44–979)		289 (41–1349)	0.123
GGT (U/L)	246 (11–1952)		287 (12–1758)	0.286
Albumin (mg/dl)	3.9 (2.1–5.1)		3.9 (2.4–5.2)	0.706
INR	1.1 (0.8–4.0)		1.1 (0.9–1.8)	0.873
BMI (kg/m^2^)	26.0 (17.0–34.0)		23.5 (16.9–29.7)	0.025
*(B)*				
N	260	276		
Gender, female/male (%)	241 (93%)/19 (7%)	97 (35%)/179 (65%)		< 0.001
Age (years)	57 (29–86)	38 (22–81)		
Age at diagnosis (years)	51 (25–83)	29 (6–70)		
IBD, none/UC/Crohn's disease/undifferentiated colitis	N.a	87/157/12/20		
Cirrhosis, (%)	114 (44%)	92 (33%)		0.224
Hemoglobin (mg/dl)	12.4 (7.0–15.7)	13.5 (8.8–17.7)		< 0.001
PLT (10^3^/µl)	166 (24–579)	240 (40–749)		< 0.001
Creatinine (mg/dl)	1.0 (1.0–4.9)	1.0 (1.0–2.5)		0.656
Bilirubin (mg/dl)	1.3 (0.2–31.2)	1.0 (0.2–45.4)		0.370
ALT (U/L)	50 (10–1244)	78 (8–1005)		< 0.001
AST (U/L)	55 (13–1543)	62 (12–542)		< 0.001
ALP (U/L)	190 (41–1318)	233 (5–1515)		< 0.000
GGT (U/L)	120 (10–4255)	200 (7–1557)		< 0.000
Albumin (mg/dl)	3.9 (2.1–5.2)	3.9 (2.1–5.6)		< 0.001
INR	1.1 (1.0–5.9)	1.0 (1.0–3.0)		0.012
BMI (kg/m^2^)	24.8 (17.3–39.0)	22.8 (15.7–36.5)		< 0.001

Analyses were performed using Mann-Whitney U test or Student *t*-test, as appropriate. Values are expressed as median (ranges), unless stated otherwise

Abbreviations: *ALP* alkaline phosphatase, *ALT* alanine aminotransferase, *AST* aspartate aminotransferase, *BMI* body mass index, *GGT* gamma-glutamyl transferase, *IBD* inflammatory bowel disease, *INR* international normalized ratio, *ND* not done, *PBC* primary biliary cholangitis, *PLT* platelet count, *PSC* primary sclerosing cholangitis, *UC* ulcerative colitis

### The *ABCB4* c.711A > T is associated with liver injury in patients with PBC

The *ABCB4* c.711A > T (rs2109505) polymorphism was successfully genotyped in all patients and controls. The exact distribution of genotypes in patients with PBC, PSC and in controls is presented in Table 2. The *ABCB4* c.711A > T risk allele was carried by a total of 833 (96.1%) patients that were included in the analysis (PSC = 95.8%, PBC = 96.3%). We did not detect any deviation from the HWE in any of the PBC or PSC cohorts (*P* > 0.05). There was no difference of genotype distribution between study patients and controls in either group (all *P* > 0.05 by Armitage’s trend test). We also did not detect differences in the distribution of this variant between patients from Szczecin and Warsaw (*P* > 0.05).

**Table 2:** Distribution of the *ABCB4* c.711A > T genotypes in patients with PBC, PSC and in controls

*ABCB4* c.711 A > T	Szczecin			Warsaw		
	[TT]	[AT]	[AA]	[TT]	[AT]	[AA]
PBC	11 (6%)	52 (26%)	133 (68%)	8 (3%)	75 (29%)	177 (68%)
PSC	5 (4%)	44 (33%)	86 (63%)	10 (4%)	71 (26%)	195 (70%)
Controls	3 (2%)	44 (29%)	103 (69%)	8 (3%)	77 (24%)	233 (73%)

Abbreviations: please see legend Table 1; *A* adenine, *ABCB4* adenosine triphosphate binding cassette subfamily B-member 4, *T* thymine

**Fig. 1:**
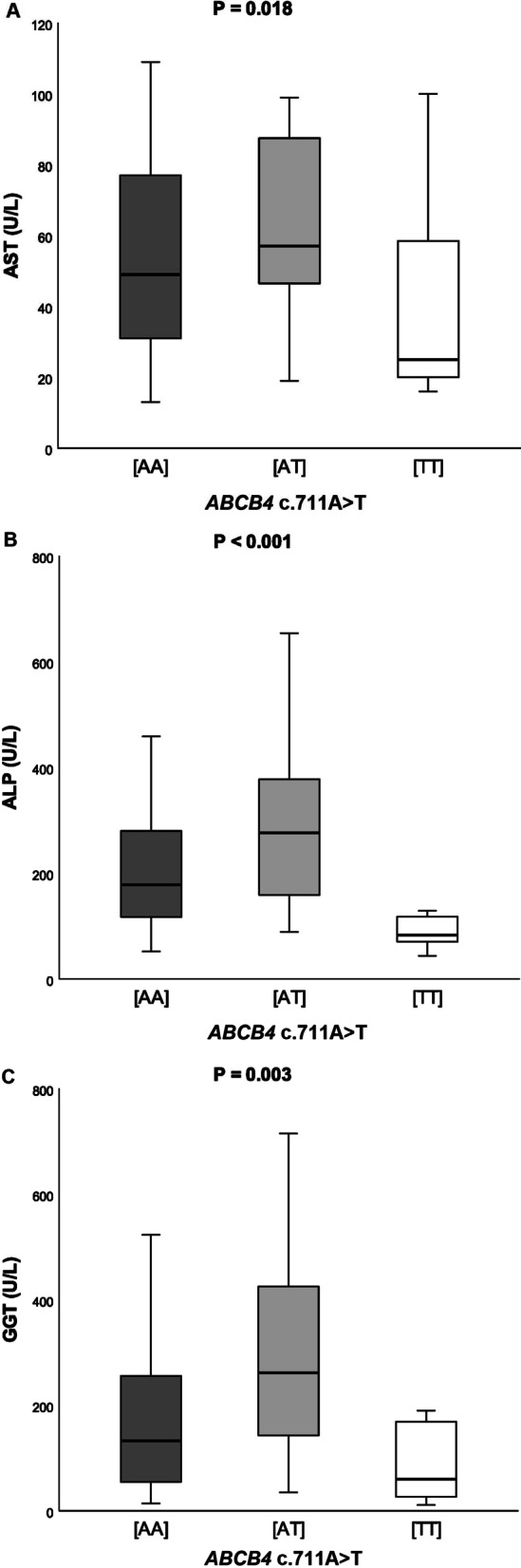
Serum AST (**A**), ALP (**B**) and GGT (**C**) in relation to the presence of the *ABCB4*c.711A > T variant in patients with PBC from Szczecin

**Fig. 2:**
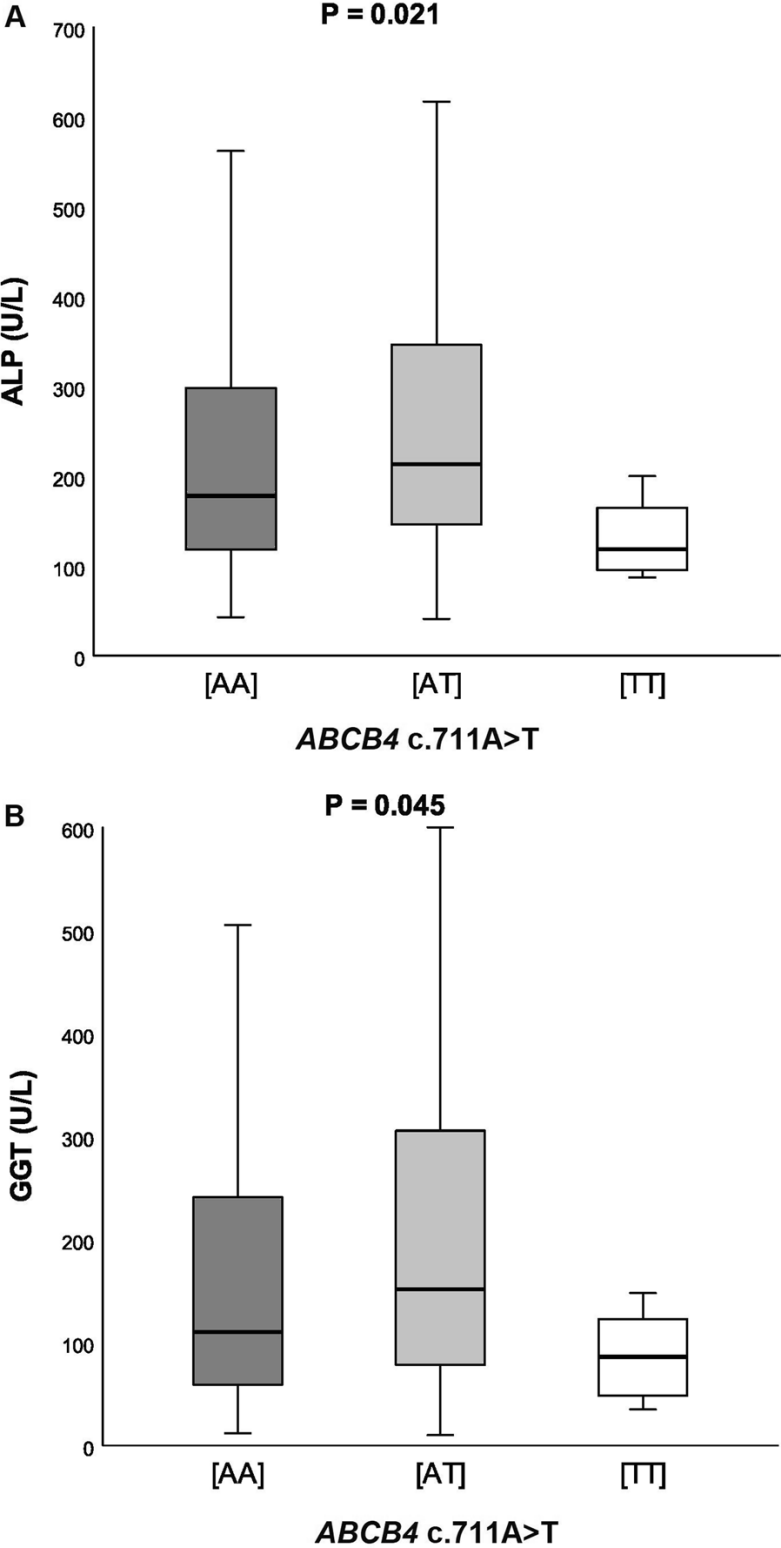
Serum ALP (**A**) and GGT (**B**) in relation to the presence of the *ABCB4* c.711A > T variant in patients with PBC from Warsaw

Subsequently, we compared the distribution of *ABCB4* variant in patients with and without liver cirrhosis separately for the PBC and PSC (Table 3). This analysis demonstrated that in both cohorts of PBC patients, carriers of the risk variant had increased odds of developing liver cirrhosis risk (Szczecin: OR = 1.84, *P* = 0.025; Warsaw: OR = 1.53, *P* = 0.039 by Armitage`s trend test). In contrast, the risk-associated allele did not increase the risk of cirrhosis in PSC patients (Table 3, all *P* > 0.05). As shown in Fig. 1, the *ABCB4* c.711A > T polymorphism modulated serum AST (*P* = 0.018, Fig. 1A), ALP (*P* < 0.001, Fig. 1B), GGT (*P* = 0.003, Fig. 1C) in the PBC patients from Szczecin and serum ALP (*P* = 0.021, Fig. 2A) and GGT (*P* = 0.045, Fig. 2B) in the PBC patients from Warsaw.

**Table 3:** Distribution of the *ABCB4* c.711A > T genotypes in patients with and without cirrhosis

		Genotype counts			Test for association		
		[TT]	[AT]	[AA]	OR	χ^2^	*P*
PBC (Szczecin)	Patients with cirrhosis	2 (3%)	15 (20%)	57 (77%)	1.84	5.00	0.025
	Patients without cirrhosis	9 (8%)	37 (30%)	76 (62%)			
PSC (Szczecin)	Patients with cirrhosis	1 (2%)	19 (36%)	33 (62%)			NS
	Patients without cirrhosis	4 (5%)	25 (30%)	53 (65%)			
PBC (Warsaw)	Patients with cirrhosis	3 (3%)	25 (22%)	86 (75%)	1.53	4.28	0.039
	Patients without cirrhosis	5 (3%)	50 (34%)	91 (62%)			
PSC (Warsaw)	Patients with cirrhosis	3 (3%)	21 (23%)	68 (74%)			NS
	Patients without cirrhosis	7 (4%)	50 (27%)	127 (69%)			

Analyses were performed using Armitage's trend test. Abbreviations: please see legend Table 1; *NS* not significant, *OR* odds ratio

We performed logistic regression analyses to investigate additional non-genetic factors for cirrhosis in our patients with PBC. As shown in Table 4A, univariate regression analysis in patients from Szczecin demonstrated significant associations of cirrhosis with both *ABCB4* c.711A > T and patients' gender. Multivariate analysis demonstrated that both the *ABCB4* c.711A > T polymorphism and patients' gender represent two independent determinants of cirrhosis in our cohort (Table 4A). In the Warsaw PBC cohort, univariate and multivariate regression analysis showed significant associations of *ABCB4* c.711A > T, as well as patients` age and gender with cirrhosis (all *P* < 0.05, Table 4B).

**Table 4:** Risk factors for developing liver cirrhosis in patients with PBC (A) Szczecin, (B) Warsaw

Factor	Univariate analysis	*P*	Multivariate analysis	*P*
	Odds ratio		Odds ratio	
*(A)*				
*ABCB4* c.711A > T	1.844	0.028	1.769	0.038
Gender	3.325	0.011	3.202	0.015
Age (years)	1.015	0.256		
BMI (kg/m^2^)	0.971	0.832		
*(B)*				
*ABCB4* c.711A > T	1.649	0.040	1.723	0.033
Gender	3.630	0.017	5.041	0.007
Age (years)	1.056	< 0.001	1.057	< 0.001
BMI (kg/m^2^)	1.022	0.503		

Analyses were performed using uni- and multivariate regression. Abbreviations: please see legend Table 1 and Table 2

**Fig. 3:**
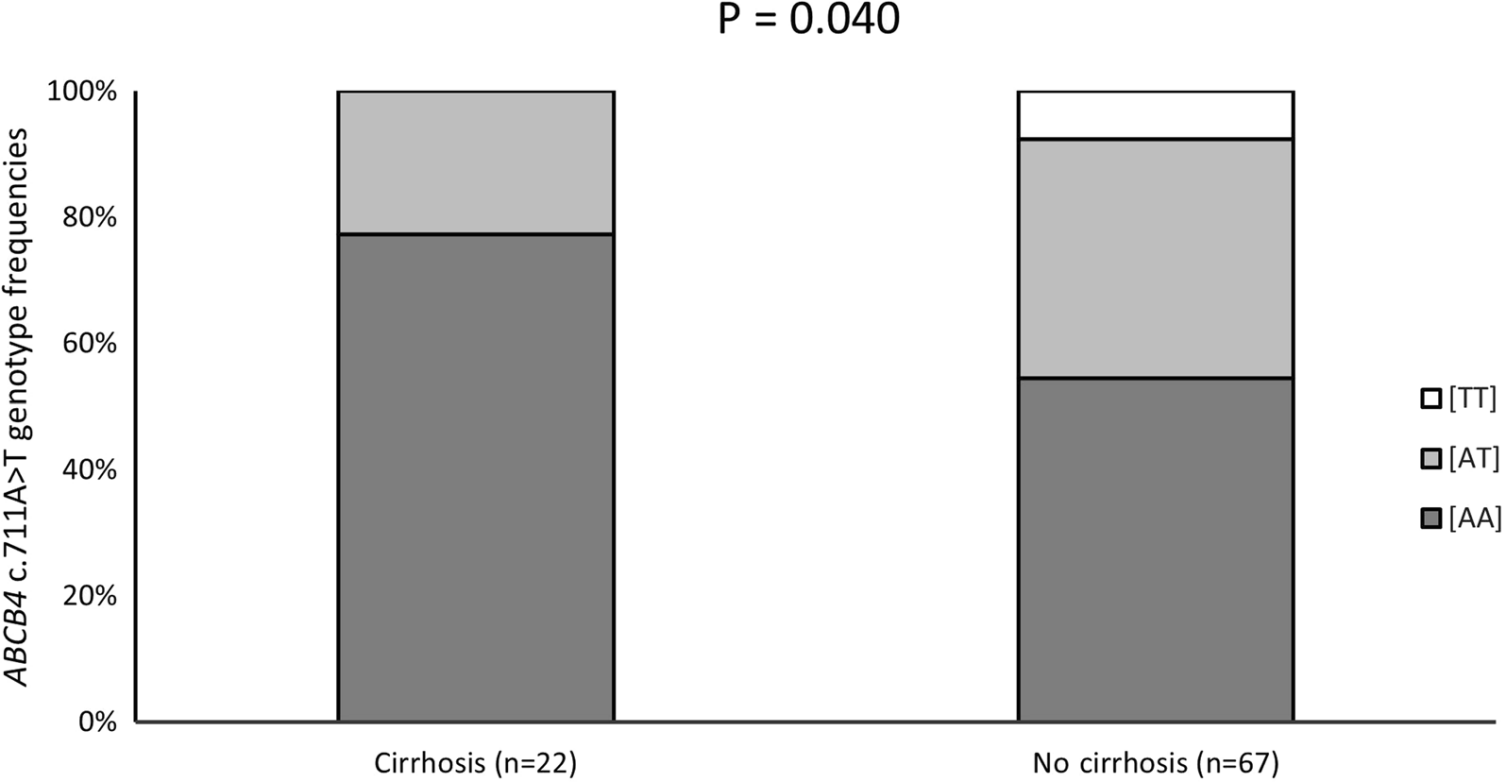
Frequencies of the *ABCB4* c.711A > T genotypes in patients with PBC from Szczecin who did and did not develop de novo cirrhosis during the follow-up period. The *ABCB4* c.711A > T risk allele was significantly more frequent in patients who developed cirrhosis

For 148 patients of the Szczecin PBC cohort, clinical data were collected at two time points with a mean follow-up of 8 ± 4 years. We detected significant improvements in laboratory findings (AST, ALT, ALP and GGT) during the follow-up (Table 5A), which was not affected by the *ABCB4* c.711A > T genotype (*P* > 0.05). On the other hand, a total of 22 patients in this cohort developed cirrhosis, and this risk was significantly modulated by the *ABCB4* c.711A > T risk variant (OR = 5.65, *P* = 0.040 by Armitage's trend test, Fig. 3).

**Table 5:** Clinical and laboratory characteristic of (A) 148 patients with PBC at two time points (follow-up: 8 ± 4 years)—Szczecin cohort, (B) 214 patients with PBC at two time points (follow-up: 4 ± 2 years)—Warsaw cohort

Variables	Subject characteristics		*P*
	At diagnosis	Follow-up	
*(A)*			
N	148		
Gender, female/male (%)	130 (88%)/18 (12%)		
Cirrhosis (%)	59 (40%)	81 (55%)	< 0.001
Bilirubin (mg/dl)	4 (0.3–40.5)	3 (0.3–29.3)	0.598
ALT (U/L)	85 (10–660)	52 (10–176)	< 0.001
AST (U/L)	84 (15–931)	64 (15–236)	0.003
ALP (U/L)	376 (37–1899)	245 (52–979)	< 0.001
GGT (U/L)	341 (17–2755)	224 (14–1952)	< 0.001
*(B)*			
N	214		
Gender, female/male (%)	199 (93%)/15 (7%)		
Cirrhosis (%)	102 (48%)	148 (69%)	< 0.001
Bilirubin (mg/dl)	1.2 (0.2–22.0)	0.75 (0.16–31.2)	0.218
ALT (U/L)	88 (11–1742)	36 (11–881)	< 0.001
AST (U/L)	86 (17–1149)	35 (9–194)	< 0.001
ALP (U/L)	369 (56–1620)	135 (42–1141)	< 0.001
GGT (U/L)	357 (34–2670)	69 (10–4255)	< 0.001

Analyses were performed using Mann-Whitney U test or Student *t*-test, as appropriate. Values are expressed as medians (ranges), unless stated otherwise. Abbreviations: please see legend Table 1

In a total of 214 PBC patients recruited in Warsaw, clinical data were collected at two time points with a mean follow-up 4 ± 2 years. During follow-up, we observed significant improvements in laboratory findings (AST, ALT, ALP and GGT, Table 5B), and 46 patients developed cirrhosis. However, in this cohort the presence of *ABCB4* c.711A > T did not modulate the risk of cirrhosis during follow-up (*P* > 0.05).

In contrast to the PBC patients, *ABCB4* c.711A > T did not modulate liver injury in patients with PSC (all *P* > 0.05). The risk of transplantation was not increased by the presence of *ABCB4* c.711A > T in either PSC or PBC patients (all *P* > 0.05). In the PSC cohort from Warsaw, recurrence of PSC after transplantation was detected in 24 patients. The *ABCB4* c.711A > T risk allele was not associated with PSC recurrence (*P* > 0.05). Although data presented recently in the literature [18] describe effects of the *ABCB4* genetic variants on patients' wellbeing, we did not detect any significant association between the *ABCB4* polymorphism and HRQoL in either PBC or PSC patients (Additional file 1:  S1 Table ).

## Discussion

Liver fibrosis and cirrhosis are the main clinical determinants of survival in patients with chronic liver disease. Previous studies [8] have demonstrated the impact of *ABCB4* variants on hepatic phenotypes [19] in patients with chronic liver diseases. The latest available data show a prevalence of PBC between 0.84 and 2.75 per 100,000 adults, and PSC between 0.1 and 4.39 per 100,000 adults in Europe [20]. Thus, the difficulty to recruit large cohorts of patients with these two rare diseases limits the power of genetic analyses. In the current study, we analysed several cohorts of patients with two chronic cholestatic conditions, which allowed us to detect the effects of *ABCB4* c.711A > T in PBC.

The *ABCB4* variant c.711A > T is very common in the general population. Indeed, 28.0% from the total of recruited patients analysed were identified as heterozygous, and 68.2% as homozygous carriers of the risk allele. Hence, the more prevalent allele represents the risk allele. As previously published, genetic polymorphisms within the *ABCB4* locus are likely to affect the function of the phospholipid transporter, predisposing carriers for cholestatic liver diseases [21, 22]. *ABCB4* c.711A > T (rs2109505) has been linked to intrahepatic cholestasis of pregnancy [15], low phospholipid-associated cholelithiasis and elevated liver serum biomarkers (ALT, AST, GGT) [16]. Ohishi et al. analysed a cohort of 148 Japanese PBC patients and reported an association of *ABCB4* c.711A > T with progression of PBC [23]. In agreement with Ohishi et al. [23], our study demonstrates that this variant confers a significant risk for the deterioration of liver function in Polish patients with PBC. This association was shown in two cohorts of patients with PBC that we recruited in Szczecin and Warsaw. In both cohorts of patients with PBC, *ABCB4* c.711A > T increased the risk of cirrhosis. Furthermore, gender was associated with increased cirrhosis risk in both groups of PBC patients, and age in the Warsaw cohort.

Of note, we did not observe any significant effects of the studied *ABCB4* polymorphism in PSC patients. This is in agreement with the different clinical presentation of patients with PBC and PSC (Table 1A, B). Overall, one might suspect that PSC has more of an autoimmune background, and therefore variants affecting bile composition are less likely to play a significant role. Hence, common variants with relatively low effect on the cholestatic phenotype, such as *ABCB4* c.711A > T, might not play a role in the progression of PSC. In our previous genetic study [24] we failed to detect effects of polymorphisms in other potential candidate modifier genes, namely *PNPLA3* or the *TM6SF2*, on liver injury in PSC although these variants were shown to affect liver scarring in other conditions [25]. Taken together, further studies are required to identify genetic modifiers in patients with PSC.

In 2020, de Vries et al. [18] used SF-36 and pruritus VAS questionnaires to analyse the quality of life in patients with *ABCB4* deficiency in comparison with PSC, PBC and the general population. This study showed impairment of life-quality in patients with *ABCB4* deficiency as compared to the general population in the energy/fatigue and general health domains [18]. Individuals with *ABCB4* deficiency and PSC differed significantly in pain domain of SF-36 whereas patients with PBC reported worse life quality in almost all domains. Low HRQoL is typical for PSC and PBC, which was previously reported by our group [26, 27]. Our current study however failed to demonstrate any associations between the *ABCB4* c.711A > T risk variant and quality of life in either PBC or PSC patients, however we cannot exclude that, other variants in this gene might affect patients well-being.

## Conclusions

The results of our study indicate that the *ABCB4* c.711A > T polymorphism significantly contributes towards fibrosis progression and increased liver injury in patients with PBC. We conclude that this variant represents a common genetic risk modifier in PBC, but not in PSC. Hence, carriers of the *ABCB4* c.711A > T risk allele might benefit from more aggressive therapy of PBC. Authors of the current EASL Clinical Practice Guidelines [28] recommend genetic analyses at the final step of clinical work-up of patients with unclear cholestatic condition. We envision, that in the future, genetic testing of patients with chronic cholestatic condition might help to stratify their risk of disease progression.

## Materials and methods

### Study cohorts

The study cohort comprised 867 adult Polish Caucasian patients with PBC and PSC prospectively recruited at Pomeranian Medical University in Szczecin and Medical University of Warsaw. The cohort from Szczecin was recruited between 2006 and 2014, and patients in Warsaw were recruited between 2016 and 2019. PBC and PSC were diagnosed according to the current guidelines of the European Association for the Study of the Liver (EASL) [28, 29]. In total, 468 Polish Caucasian subjects (healthy blood donors and outpatients without liver diseases) served as controls. The first control group consisted of blood donors without signs of acute or chronic liver diseases recruited in Szczecin. The second control group was recruited at the outpatient clinic of the University Hospital in Warsaw. This cohort comprised patients without liver diseases who were treated in specialist outpatient clinics. The study protocol follows the ethical guidelines of the declaration of Helsinki, and was approved by the local ethics committee (BN-001/43/06, KB/58/A/2016). Informed consent was obtained from all patients and controls. The diagnosis of PBC and PSC was based on the EASL criteria [28–30]. All PBC and PSC patients were treated with ursodeoxycholic acid. None of the studied patients received obeticholic acid (OCA). Also, none of the patients received bezafibrate. Clinical data were collected at two time points: at the moment of diagnosis and the last clinical visit. Venous blood samples were drawn from all patients for DNA genotyping and for all clinical analyses, including serum levels of liver-related biomarkers. The presence of liver cirrhosis was established either by liver biopsy or clinical signs of liver images (abdominal ultrasonography or computer tomography) showing structural features of cirrhosis and/or symptoms of portal hypertension such as ascites, splenomegaly, esophageal/gastric varices. These were supported by a typical biochemistry including decrease of platelets count.

The generic Medical Outcomes Study Short Form (SF-36) and the disease-specific PBC-40 questionnaires were used for assessment at health related quality of life. The SF-36 questionnaire contains 36 items grouped into 8 domains of physical (Physical Functioning, Role limitation—Physical, Bodily Pain and General Health) and mental health (Vitality, Social Functioning, Role limitation—Emotional, and Mental Health). Scores can be gathered into 2 summary scores—a Mental Component Summary score and Physical Component Summary score. The PBC-40 questionnaire contains 40 questions grouped into 5 (Other symptoms, Itch, Fatigue, Cognitive, Social and Emotional) domains.

### Genotyping

The DNA for genotyping of the *ABCB4* c.711A > T (p.I237I, rs2109505) polymorphism was extracted from EDTA-anticoagulated blood using DNAasy Blood & Tissue Kit (Qiagen, Hilden, Germany). The genotyping was performed using TaqMan SNP Genotyping Assays (Applied Biosystems, Foster City, USA).

### Statistical analysis

Data were presented as medians (and ranges) for continuous variables. The genotype frequencies of the *ABCB4* c.711A > T polymorphism were tested for consistency with the Hardy–Weinberg equilibrium (HWE). Differences of allele and genotype frequencies were analysed by χ^2^ and Armitage’s trend tests, respectively (https://ihg.helmholtz-muenchen.de/cgi-bin/hw/hwa1.pl). The Kolmogorov–Smirnov test was used to test normality of the distributions in the study cohort. The relation between the *ABCB4* c.711A > T variant and baseline patient characteristics were tested by Mann–Whitney U test or Student *t*-tests, as appropriate. Follow-up data were analyzed using paired samples *t*-test. Univariate and multivariate logistic regression analyses were used to test the effects of factors such as *ABCB4* variant, age, BMI and gender on cirrhosis. Data analysis was performed using SPSS software (v.26.0, IBM, Armonk, New York, USA). A *P* value < 0.05 was regarded as significant.

## Supplementary Information

**Additional file 1.** **S1 Table.** A. Health-related quality of life (HRQoL) in patients with PBC in relation to the ABCB4 genotype (Szczecin). B. Health-related quality of life (HRQoL) in patients with PBC in relation to the ABCB4 genotype (Warsaw).

## Data Availability

Data from patients who have consented to share their data with other investigators are available upon request from the corresponding author.

## Abbreviations

A: Adenine
ABCB4: Adenosine triphosphate binding cassette subfamily B-member 4
ALP: Alkaline phosphatase
ALT: Alanine aminotransferase
AST: Aspartate aminotransferase
BMI: Body mass index
GGT: Gamma-glutamyl transferase
HRQoL: Health-related quality of life
HWE: Hardy–Weinberg equilibrium
ICP: Intrahepatic cholestasis of pregnancy
INR: International normalized ratio
PBC: Primary biliary cholangitis
PBC-40: Disease specific questionnaire
PFIC-3: Progressive familial intrahepatic cholestasis type 3
PLT: Platelet count
PSC: Primary sclerosing cholangitis
SF-36: Short form health survey
SNP: Single nucleotide polymorphism
T: Thymine
VAS: Visual analogue scale
