# Supplementary material for: A common variant in the hepatobiliary phospholipid transporter ABCB4 modulates liver injury in PBC but not in PSC: prospective analysis in 867 patients
Source: Orphanet J Rare Dis. 2022 Nov 17;17:419. doi: 10.1186/s13023-022-02565-6 (PMC9670364; doi:10.1186/s13023-022-02565-6)
Supplement: Supplementary file 1 — Additional file 1. S1 Table. A. Health-related quality of life (HRQoL) in patients with PBC in relation to the ABCB4 genotype (Szczecin). B. Health-related quality of life (HRQoL) in patients with PBC in relation to the ABCB4 genotype (Warsaw). [file 13023_2022_2565_MOESM1_ESM.docx]

**S1 Table.**

**A. Health-related quality of life (HRQoL) in patients with PBC in relation to the *ABCB4* genotype (Szczecin).**

| **HRQoL domain** | **HRQoL measures** | **P** |
| --- | --- | --- |
| **PBC-40** |  |  |
| *Other symptoms* | 17 (7 - 28) | 0.066 |
| *Itch* | 5 (3 - 15) | 0.233 |
| *Fatigue* | 29 (11 - 54) | 0.938 |
| *Cognitive* | 13 (6 - 26) | 0.709 |
| *Social and emotional* | 30 (13 - 54) | 0.539 |
| **SF-36** |  |  |
| *Physical Functioning* | 61 (0 - 100) | 0.713 |
| *Role limitation-Physical* | 39 (0 -100) | 0.728 |
| *Bodily Pain* | 57 (0 - 100) | 0.161 |
| *General Health* | 46 (0 - 95) | 0.454 |
| *Vitality* | 48 (5 - 100) | 0.946 |
| *Social Functioning* | 65 (0 - 100) | 0.767 |
| *Role limitation-Emotional* | 51 (0 - 100) | 0.312 |
| *Mental Health* | 60 (0 - 100) | 0.927 |
| *Physical Component Summary score* | 51 (6 - 96) | 0.477 |
| *Mental Component Summary score* | 56 (8 - 100) | 0.387 |

**B. Health-related quality of life (HRQoL) in patients with PBC in relation to the *ABCB4* genotype (Warsaw).**

| **HRQoL domain** | **HRQoL measures** | **P** |
| --- | --- | --- |
| **PBC-40** |  |  |
| *Other symptoms* | 17 (7 - 28) | 0.829 |
| *Itch* | 6 (3 - 15) | 0.546 |
| *Fatigue* | 31 (11 - 53) | 0.458 |
| *Cognitive* | 14 (6 - 30) | 0.668 |
| *Social and emotional* | 34 (13 - 60) | 0.739 |
| **SF-36** |  |  |
| *Physical Functioning* | 59 (0 - 100) | 0.457 |
| *Role limitation-Physical* | 36 (0 -100) | 0.596 |
| *Bodily Pain* | 56 (0 - 100) | 0.849 |
| *General Health* | 42 (0 - 90) | 0.356 |
| *Vitality* | 44 (5 - 100) | 0.822 |
| *Social Functioning* | 58 (0 - 100) | 0.364 |
| *Role limitation-Emotional* | 51 (0 - 100) | 0.370 |
| *Mental Health* | 58 (0 - 100) | 0.917 |
| *Physical Component Summary score* | 48 (3 - 95) | 0.890 |
| *Mental Component Summary score* | 53 (1 - 100) | 0.488 |

Data are presented as median (range). Abbreviations: HRQoL, Health-related quality of life; PBC-40, disease specific questionnaire; SF-36, short form health survey.
